# The effects of conjugated linoleic acid supplementation on glycemic control, adipokines, cytokines, malondialdehyde and liver function enzymes in patients at risk of cardiovascular disease: a GRADE-assessed systematic review and dose–response meta-analysis

**DOI:** 10.1186/s12937-023-00876-3

**Published:** 2023-10-05

**Authors:** Nasim Ghodoosi, Niloufar Rasaei, Kian Goudarzi, Maral Hashemzadeh, Sina Dolatshahi, Hossein Salehi Omran, Niusha Amirani, Damoon Ashtary-larky, Ghazaleh Shimi, Omid Asbaghi

**Affiliations:** 1 Department of Community Nutrition, School of Nutritional Sciences and Dietetics, Tehran University of Medical Sciences (TUMS), Tehran, Iran; 2 Faculty of Medicine, Shahid Beheshti University of Medical Sciences, Tehran, Iran; 3 Department of Community Nutrition, School of Nutrition and Food Sciences, Shiraz University of Medical Sciences, Shiraz, Iran; 4 Faculty of Medicine, Alborz University of Medical Sciences, Karaj, Iran; 5 Nutrition and Metabolic Diseases Research Center, Ahvaz Jundishapur University of Medical Sciences, Ahvaz, Iran; 6 Department of Cellular and Molecular Nutrition, Faculty of Nutrition Science and Food Technology, National Nutrition and Food Technology Research Institute, Shahid Beheshti University of Medical Sciences, Tehran, Iran; 7 Cancer Research Center, Shahid Beheshti University of Medical Sciences, Tehran, Iran; 8 Student Research Committee, Shahid Beheshti University of Medical Sciences, Tehran, Iran

**Keywords:** Conjugated linoleic acid, Cytokine, Adipokine, Glycemic profile, Meta-analysis

## Abstract

**Background:**

The present systematic review and meta-analysis sought to evaluate the effects of conjugated linoleic acid (CLA) supplementation on glycemic control, adipokines, cytokines, malondialdehyde (MDA) and liver function enzymes in patients at risk of cardiovascular disease.

**Methods:**

Relevant studies were obtained by searching the PubMed, SCOPUS and Web of Science databases (from inception to January 2023). Weighted mean differences (WMD) and 95% confidence intervals (CIs) were pooled using a random-effects model. Heterogeneity, sensitivity analysis, and publication bias were reported using standard methods.

**Results:**

A pooled analysis of 13 randomized controlled trials (RCTs) revealed that CLA supplementation led to a significant increment in fasting blood glucose (FBG) (WMD: 4.49 mg/dL; 95%CI: 2.39 to 6.59; *P* < 0.001), and aspartate aminotransferase (AST) (WMD: 2.54 IU/L; 95%CI: 0.06 to 5.01; *P* = 0.044). Moreover, CLA supplementation decreased leptin (WMD: -1.69 ng/ml; 95% CI: -1.80 to -1.58; *P* < 0.001), and interleukin 6 (IL-6) (WMD: -0.44 pg/ml; 95%CI: -0.86 to -0.02; *P* = 0.037). However, there was no effect on hemoglobin A1c (HbA1c), homeostatic model assessment for insulin resistance (HOMA-IR), C-reactive protein (CRP), tumor necrosis factor alpha (TNF-α), and alanine aminotransferase (ALT) adiponectin compared to the control group.

**Conclusion:**

Our findings showed the overall favorable effect of CLA supplementation on the adipokines and cytokines including serum IL-6, and leptin, while increasing FBG and AST. It should be noted that the mentioned metabolic effects of CLA consumption were small and may not reach clinical importance.

**Prospero registeration cod:**

CRD42023426374.

**Supplementary Information:**

The online version contains supplementary material available at 10.1186/s12937-023-00876-3.

## Introduction

Cardiovascular diseases (CVDs) create huge morbidity and mortality risks worldwide. They place a significant economic burden on the healthcare system. Unhealthy lifestyles like obesity, alcohol consumption, unhealthy diet, and physical inactivity are traditional risk factors of CVDs. Other risk factors linked to CVDs are genetic predisposition and presence of chronic diseases [1]. Diabetes is one well-established example. Based on studies, controlling the glycemic profile may be beneficial in preventing CVD events, by decreasing oxidative stress and vascular complications [2]. Deregulation of adipokines which is linked to obesity can cause a low-grade, chronic inflammatory state that may develop CVDs [3]. Pro-inflammatory cytokines and a parameter of oxidative stress, MDA, are also biomarkers for predicting the risk of CVDs [4, 5]. Moreover, CVDs are associated with the accumulation of liver fat and increased levels of the liver enzymes [6]. Therefore, effective strategies are highly needed for treating high-risk people for CVDs to help reduce the complications.

Among different possible alternative strategies to prevent CVDs (medical therapy, surgical treatments, and dietary supplements), nutraceuticals have gained public interest [7]. One nutraceutical which may have a role in modulating CVD risks is conjugated linoleic acid (CLA). CLA is an omega-6 polyunsaturated fatty acid found mostly in meat and dairy products. It is a family of positional and geometric isomers of linoleic acid. Cis-9, trans-11 and trans-10, cis-12 are major isomers of CLA in food [8, 9]. Some effects of CLA can be isomer-specific and difference in the intake of CLA isomers may influence the results of studies conducting on CLA. Thus, dietary supplementation of CLA, with different isomer ratios, has drawn the attention of researchers in healthcare systems.

The relationship between CLA consumption and glycemic profile still needs to be clarified. Eight weeks of supplementation with CLA showed efficacy of this supplement in decreasing body weight in individuals with insulin resistance [10]. A study working on obese children without diabetes revealed that CLA improved fasting insulin and homeostatic model assessment for insulin resistance (HOMA-IR)[11]. A meta-analysis on 32 randomized controlled trials (RCTs) indicated no effects of CLA consumption on fasting blood glucose (FBG) [12]. Furthermore, supplementing with trans-10,cis-12 isomer of CLA for 12 weeks increased insulin resistance and fasting glucose in abdominally obese men [13]. Consumption of another active isomer of CLA (cis-9,trans-11) for three months also increased insulin resistance in abdominally obese men [14].

CLA seems to elevate C-reactive protein (CRP) levels [15–18]. However, CLA effects on inflammatory cytokines (tumor necrosis factor alpha (TNF-α) and **i**nterleukin 6 (IL-6)) and adipokines (adiponectin and leptin) remain unanswered. In some meta-analyses CLA consumption increased TNF-α [17], decreased TNF-α and IL-6 [15, 17], and caused no changes in IL-6 [18]. Decreasing effect of CLA consumption on circulating leptin was observed in one study [19], while in another study no effect was shown [15]. In a meta-analysis conducted by Rastgoo et al. (2023), CLA supplementation did not change adiponectin, but Mazidi et al. (2017) showed a significant reduction effect of CLA on adiponectin levels [18].

Results of the efficacy of CLA on liver enzymes and MDA are inconclusive. No changes of aspartate aminotransferase (AST) / alanine aminotransferase (ALT) activation were observed after 12 weeks of CLA intake in obese and overweight women [20]. However, a meta-analysis analyzing 13 RCTs, CLA increased AST significantly and ALT non-significantly [21]. A meta-analysis of 11 trials indicated that intervention with CLA could not change malondialdehyde (MDA) [22]. Conversely, another meta-analysis, also including 11 RCTs, showed that CLA supplementation decreased MDA levels, significantly [23]. Interestingly, another meta-analysis conducted by Haghighat et al. (2022) proposed that CLA may increase AST/ ALT and reduce MDA levels or cause no change [24].

Consequently, to detect the inconsistency, the present systematic review and meta-analysis aimed to update previous meta-analyses and include all subsequent trials that investigated the effects of CLA supplementation on glycemic control, adipokine, cytokine, MDA, and liver function enzymes in patients at risk of cardiovascular diseases.

## Materials and methods

### Search strategy and study selection

To conduct this study, the protocol of Preferred Reporting Items for Systematic Reviews and Meta-Analyzes (PRISMA) was selected between the various methods for reporting systematic reviews and meta-analyses [25]. The literature was searched comprehensively in the various online databases, including PubMed, Scopus, and ISI Web of Science, to find relevant studies without any date or language limitation up to January 2023. Therefore, the following search terms in titles and abstracts were searched **(**supplementary file 1). Moreover, the Google scholar database was searched manually. The Endnote software was applied as a screening tool for included studies. Search strategy and study selection were conducted by two separate investigators.

### Eligibility criteria

All studies with the following features were included in this meta-analysis: 1) randomized controlled trials (RCTs) that evaluated the effects of CLA supplementation on these factors as an outcome ( FBG, Insulin, HbA1c, HOMA-IR, CRP, TNF-α, IL-6, leptin, adiponectin, AST, ALT), with a control group, 2) studies conducted on adults (≥ 18 years), 3) studies used CLA supplementation as an intervention, 4) studies with parallel or crossover designs, 5) studies with outcome reporting at the beginning and the end of the intervention, 6) studies conducted on subjects at risk of CVDs (being over-weight and obese, having metabolic syndrome, type 2 diabetes mellitus, hypertension, and hyperlipidemia, atherosclerotic patients and non-alcoholic fatty liver disease).

### Exclusion criteria

By analyzing the full text of the articles, the following studies were excluded: 1) animal, review, ecological, and observational studies, 2) studies conducted on individuals younger than 18 years, 3) studies without randomization or placebo or control groups, 4) studies conducted on healthy individuals.

### Data extraction

Records were screened primarily by two separate investigators following the title and abstract assessment to detect eligibility. Next, to determine if the potential studies could be included in the study, their full texts were reviewed closely. Ultimately, the following data were extracted: the name of the first author, the year of the publication, the location of the study, the study design, the sample size in each group, the characteristics of the subjects such as mean age, sex, body mass index (BMI), health status, the doses of CLA used for the intervention, the duration of the interventions, the mean changes, and the standard deviation (SD) of the markers throughout the study, for both intervention and control groups. By observing multiple data at various time points for a specific study, the most recent was considered.

### Quality assessment

The quality assessment of the qualified studies was performed by two separate investigators using the Cochran scoring method [26]. It possessed seven criteria to evaluate the risk of bias, which are as follows: random sequence generation, allocation concealment, blinding of participants and personnel, blinding of outcome assessment, incomplete outcome data, selective reporting, and other biases. Consequently, terms such as “Low”, “High”, or “Unclear” were used to assess each field. In addition, any dissimilarity was clarified by the corresponding authors.

### Data synthesis and statistical analysis

In this meta-analysis, to detect the overall effect sizes, weighted mean differences (WMD) and the SDs of measures from both intervention and control groups were extracted using the random-effects model, according to DerSimonian And Laird method [27]. Furthermore, without meaningful changes reporting, it was calculated by using this formula: mean change = final values − baseline values, and SD changes were calculated by the following formula [28]:

$$\mathbf{S}\mathbf{D}\,\mathbf{c}\mathbf{h}\mathbf{a}\mathbf{n}\mathbf{g}\mathbf{e}=\sqrt{[(\mathbf{S}\mathbf{D}\,\mathbf{b}\mathbf{a}\mathbf{s}\mathbf{e}\mathbf{l}\mathbf{i}\mathbf{n}\mathbf{e})^2+(\mathbf{S}\mathbf{D}\,\mathbf{f}\mathbf{i}\mathbf{n}\mathbf{a}\mathbf{l})^2-(2\mathbf{R}\times \mathbf{S}\mathbf{D}\,\mathbf{b}\mathbf{a}\mathbf{s}\mathbf{e}\mathbf{l}\mathbf{i}\mathbf{n}\mathbf{e}\times \mathbf{S}\mathbf{D}\,\mathbf{f}\mathbf{i}\mathbf{n}\mathbf{a}\mathbf{l})}$$

We considered the correlation coefficient (R) to be 0.8. We also converted standard errors (SEs), 95% confidence intervals (CIs), and interquartile ranges (IQRs) to SDs by applying the Hozo et al. method [29]. To consider between-study variations the random-effects model was used to determine the overall effect size. The Between-study heterogeneity was also tested by Cochran's Q test and was measured by the I-squared statistic (I^2^) [30]. I2 > 40% or *p*-value < 0.05 was considered high between-study heterogeneity. To detect potential sources of heterogeneity [31], subgroup analyses were carried out following the pre-planned criteria, including study duration (≤ 16 and > 8 weeks), baseline levels of FBG, Insulin, HbA1c, HOMA-IR, CRP, TNF-α, IL-6, leptin, adiponectin, AST, ALT, baseline BMI, sex (male, female, both), health status (Metabolic syndrome, Type2 Diabetes, Hyperlipidemia, Hypertension, Non-Alcoholic Fatty Liver Disease (NAFLD)) and intervention doses (mg/d). We conducted a sensitivity analysis to determine the effect of each specific study on the overall estimation [32]. The possibility of publication bias was tested using Egger's regression test and the visually inspected funnel plot test [33]. STATA, version 11.2 (Stata Corp, College Station, TX) was used to carry out statistical analyses. The *p*-values < 0.05 were considered statistically significant in all analyses.

## Results

### Study selection

**Fig. 1:**
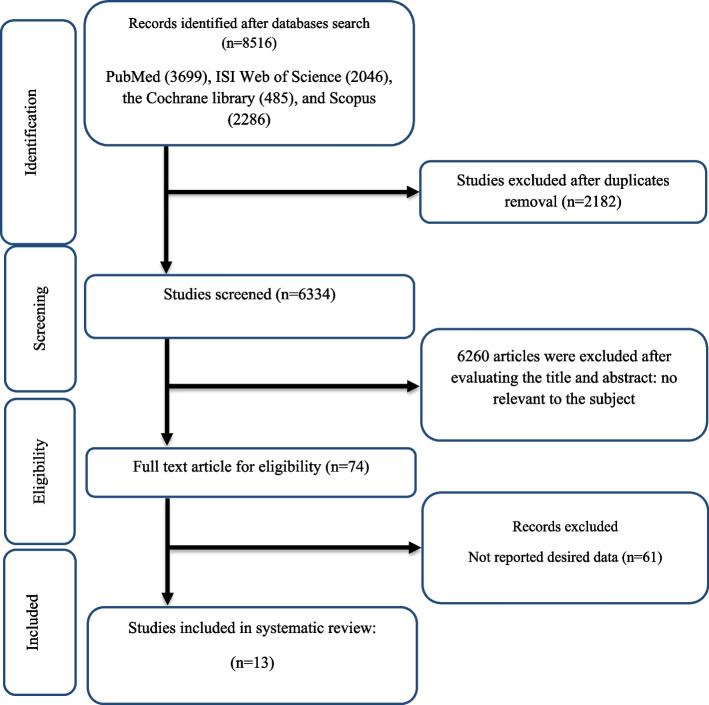
Flow chart of study selection for inclusion trials in the systematic review

As mentioned in Fig. 1, 8516 studies were found in online databases at the first step of the search protocol. As a result, 2182 studies were duplicates and were subsequently removed. Afterward, the titles and abstracts of the studies were assessed exhaustively, and 6260 unrelated studies were deleted. Furthermore, irrelevant studies based on inclusion criteria, review, and animal studies were excluded. Moreover, we removed 61 studies without necessary data reporting by executing a comprehensive full-text assessment. After all, this study included 13 appropriate studies with the closest characteristics to the mentioned inclusion criteria.

### Study characteristic

Finally, we qualified and included 13 studies, with 723 overall participants (348 cases and 375 controls). 2002 until 2018 was the publication date of all included studies. The intervention duration in qualified articles differed from 8 [34–41] to 16 [42] weeks. The sample size varied from 14 [43] to 80 [36] individuals. Parallel [35–46] and crossover RCTs [34] were the designs of qualified studies. Various subjects participated in included studies, like obese men with metabolic syndrome [44], type 2 diabetes mellitus patients [35, 37, 38, 46], overweight subjects with low-density lipoprotein phenotype B [45], Obesity-related hypertensive patients [36], postmenopausal women with type 2 diabetes mellitus [42], overweight hyperlipidemic individuals [34], atherosclerotic patients [41], patients with metabolic syndrome [43], and non-alcoholic fatty liver disease patients [39, 40]. All studies were executed in the UK [35], Iran [37–41], Netherlands [45], Canada [34], Sweden [44], Germany[42], Brazil [43], France [46], and China [36]. In included investigations, two studies were performed on just females [42, 43], four studies on males [34, 44], and the others were carried out on both [35–41, 45, 46]. The features of included studies are mentioned in Table 1.

**Table 1:** Characteristic of included studies in meta-analysis

**studies**	**Country**	**Study Design**	**Participant**	**Sample size and Sex**	**Sample size**		**Trial Duration** **(Week)**	**Means Age**		**Means BMI**		**Intervention**	
					**IG**	**CG**		**IG**	**CG**	**IG**	**CG**	**CLA (g/d)**	**Control group**
**RISerus et al. 2002 (a) **[44]	Sweden	paralell, R, PC, DB	Obese Men with the Metabolic Syndrome	M: 38	19	19	12	51 ± 7.1	53 ± 10.1	30.1 ± 1.8	30.2 ± 1.8	3.4	placebo
**RISerus et al. 2002 (b)**	Sweden	paralell, R, PC, DB	Obese Men with the Metabolic Syndrome	M: 38	19	19	12	55 ± 7.1	53 ± 10.1	31.2 ± 2.5	30.2 ± 1.8	3.4	placebo
Moloney et al. 2004 [35]	United Kingdom	paralell, R, PC, DB	type 2 diabetes mellitus	M/F: 32	16	16	8	63.8 ± 8.8	58.1 ± 10.8	29.1 ± 4	30.7 ± 4.8	3	control diet
Naumann et al. 2006 (a) [45]	Netherlands	paralell, R, PC, DB	overweight subjects with LDL phenotype B	M/F: 68	34	34	13	51 ± 7	51 ± 9	28.6 ± 2.3	28 ± 2.2	3	control diet
**Naumann et al. 2006 (b)**	Netherlands	paralell, R, PC, DB	overweight subjects with LDL phenotype B	M/F:53	19	34	13	55 ± 7	51 ± 9	29.3 ± 2.4	28 ± 2.2	3	control diet
Schmitt et al. 2006 [46]	France	paralell, R, PC, DB	type 2 diabetes	M/F (F:10, M:16)	13	13	12	54.38 ± 8.96	61.62 ± 9.27	32.07 ± 5.37	31.81 ± 4.16	4.5	control diet
Zhao et al. 2009 [36]	China	paralell, R, PC, DB	Obesity-Related Hypertension	M/F (F:36, M:44)	40	40	8	62.3 ± 3.5	59.4 ± 2.4	32.3 ± 2.3	31.2 ± 1.4	4.5	control diet
Norris et al. 2009 [42]	Germany	paralell, R, PC, DB	postmenopausal women with type 2 diabetes mellitus	F: 55	22	33	16	59.4 ± 7.3	60.1 ± 7.3	37.1 ± 7.2	36.3 ± 6.1	6.4	control diet
Shadman et al. 2010 [37]	Iran	paralell, R, PC, DB	type 2 diabetic patients	M/F (F:20, M:19)	19	20	8	45.14 ± 5.77	46.53 ± 4.38	27.48 ± 3.59	27.13 ± 1.87	3	placebo
Joseph et al. 2011 (a) [34]	Canada	crossover, R, PC, DB	Overweight, Hyperlipidemic	M: 27	27	27	8	18–60	18–60	31.5 ± 4	31.3 ± 4	3.5	placebo
**Joseph et al. 2011 (b)**	Canada	crossover, R, PC, DB	Overweight, Hyperlipidemic	M: 27	27	27	8	18–60	18–60	31.4 ± 4	31.3 ± 4	3.5	placebo
Shadman et al. 2013 [38]	Iran	paralell, R, PC, DB	overweight type2 diabetics	M/F (F:21, M:18)	19	20	8	45.1 ± 5.7	45.5 ± 4.3	27.4 ± 0.5	27.1 ± 1.8	3	placebo
**Carvalho et al. 2013**	Brazil	paralell, R, PC, DB	metabolic syndrome	F: 14	7	7	12	40 ± 14.12	42 ± 5.16	32.53 ± 2.1	32.3 ± 2.16	3	placebo
Eftekhari et al. 2013 [41]	Iran	paralell, R, PC	atherosclerotic patients	M/F: 57	29	28	8	52.79 ± 14.11	55.85 ± 14.13	24.02 ± 2.76	24.66 ± 2.34	3	control diet
Ebrahimi-Mameghani et al. 2016 [40]	Iran	paralell, R, PC, B	Non-Alcoholic Fatty Liver Disease	M/F (F:33, M:5)	19	19	8	36.74 ± 6.87	38.58 ± 8.24	32.72 ± 4.63	35.27 ± 3.46	3	placebo
Abedi et al. 2018 [39]	Iran	paralell, R, PC, SB	Non-Alcoholic Fatty Liver Disease	M/F (F:32, M: 6)	19	19	8	36.74 ± 6.87	38.58 ± 8.24	32.72 ± 4.63	35.27 ± 3.46	3	control diet

Abbreviations: *IG* Intervention group, *CG* Control group, *DB* Double-blinded, *SB* Single-blinded, *PC* Placebo-controlled, *CO* Controlled, *RA* Randomized, *NR* Not reported, *F* Female, *M* Male, *NR* Not reported

### Quality assessment

By assessing the general risk of bias, five studies acquired a moderate risk of bias [35, 38, 41, 45, 46], two studies showed a low risk of bias [40, 42], and five studies mentioned a high risk of bias [34, 36, 37, 39, 44] (Table 2).

**Table 2:** Risk of bias assessment using the Cochran scoring method

Study	Random sequence generation	Allocation concealment	Selective reporting	Other sources of bias	Blinding (participants and personnel)	Blinding (outcome assessment)	Incomplete outcome data	General risk of bias
RISerus et al. 2002 [44]	L	H	H	L	L	U	H	High
Moloney et al. 2004 [35]	L	H	H	L	L	U	L	Moderate
Naumann et al. 2006 [45]	L	H	H	L	L	U	L	Moderate
Schmitt et al. 2006 [46]	L	H	H	L	L	U	L	Moderate
Zhao et al. 2009 [36]	L	H	H	H	L	U	L	High
Norris et al. 2009 [42]	L	L	H	L	L	U	L	Low
Shadman et al. 2010 [37]	L	H	H	H	L	L	L	High
Joseph et al. 2011 [34]	L	H	H	H	L	U	H	High
Shadman et al. 2013 [38]	L	H	H	L	L	U	L	Moderate
Carvalho et al. 2013	L	H	H	H	L	U	L	High
Eftekhari et al. 2013 [41]	L	L	H	H	L	U	L	Moderate
Ebrahimi-Mameghani et al. 2016 [40]	L	L	H	L	L	L	L	Low
Abedi et al. 2018 [39]	L	L	H	L	H	H	L	High

*L* Low risk of bias, *H* High risk of bias, *U* Unclear risk of bias

General Low risk < 2 high risk

General moderate risk = high risk

General high risk > 2 high ris

## Meta-analysis

### Effect of CLA on FBG, fasting insulin, HbA1c, and HOMA-IR

**Fig. 2:**
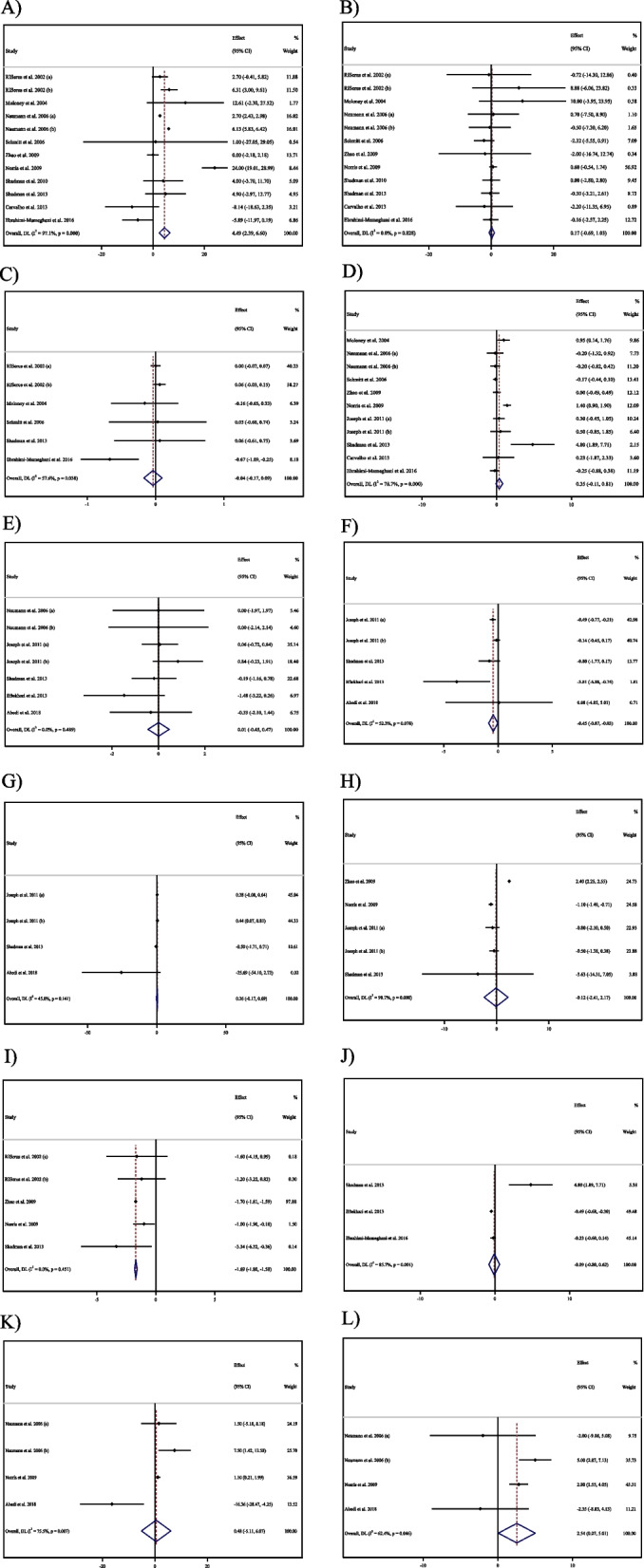
Forest plot detailing weighted mean difference and 95% confidence intervals (CIs) for the effect of CLA supplementation on **A** FBG (mg/dl); **B** Insulin (pmol/l); **C** HbA1c (%); **D** HOMA-IR; **E** CRP (mg/l); **F** IL-6 (pg/ml); **G** TNF-α (pg/ml); **H** Adiponectin (ng/ml); **I** Leptin (ng/ml); **J** MDA (umol/l); **K** ALT (U/L); and **L**) AST (U/L)

Assessing 12 overall effect sizes from 10 studies for FBG and fasting insulin, and six effect sizes from five studies for HbA1c, revealed that CLA supplementation failed to affect HbA1c and fasting insulin levels significantly (for HbA1c WMD: -0.03%; 95%CI: -0.17 to 0.09; *P* = 0.567) (Fig. 2C), (for fasting Insulin WMD: 0.16 mU/L; 95%CI: -0.69 to 1.02; *P* = 0.702) (Fig. 2B), whereas it made a significant increasing effect on FBG levels (for FBG WMD: 4.49 mg/dL; 95%CI: 2.39 to 6.59; *P* < 0.001) (Fig. 2A). We also observed high heterogeneity for FBG (I^2^ = 97.1%), moderate for HbA1c (HbA1c I^2^ = 57.6%), and no heterogeneity for insulin among studies (I^2^ = 0.0%). Additionally, subgroup analysis indicated that CLA supplementation increased FBG levels in the long-term intervention (≥ 12 weeks), in lower doses (< 3g), among overweight (25 < BMI < 29.9) or hyperlipidemic individuals, and in studies conducted on participants with higher baseline levels of FBG (≥ 100). Moreover, in NAFLD patients, CLA supplementation significantly lowered HbA1c levels. Evaluating 11 overall effect sizes from nine studies demonstrated that CLA supplementation failed to alter HOMA-IR (for HOMA-IR WMD: 0.34; 95%CI: -0.11 to 0.81; *P* = 0.140) (Fig. 2D). In addition, a significant degree of between-studies heterogeneity was observed (I^2^ = 78.7%). Moreover, subgroup analysis indicated that CLA supplementation increased HOMA-IR in female participants (Table 3).

**Table 3:** Subgroup analyses of CLA supplementation on glycemic control, adipokine, cytokine, malondialdehyde and liver function enzymes in subjects with metabolic disorders

	Number of effect size	WMD (95%CI)	*P*-value	heterogeneity		
				P heterogeneity	I^2^	P between sub-groups
Subgroup analyses of CLA on serum FBG (mg/dL)						
Overall effect	12	4.49 (2.39, 6.59)	** < 0.001**	< 0.001	97.1%	
Baseline FBG (mg/dl)						
≥ 100	9	5.90 (1.40, 10.40)	**0.010**	< 0.001	90.5%	0.205
< 100	3	1.13 (-4.70, 6.96)	0.704	< 0.001	94.6%	
Trial duration (week)						
≥ 12	7	6.24 (3.71, 8.77)	** < 0.001**	< 0.001	98.3%	0.040
<12	5	1.07 (-3.17, 5.32)	0.620	0.065	54.8%	
Intervention dose (g/day)						
≥ 3	5	7.57 (-0.17, 15.33)	0.055	< 0.001	94.8%	0.247
<3	7	2.73 (0.04, 5.42)	**0.046**	< 0.001	98.0%	
Baselin BMI (kg/m^2^)						
Obese (> 30)	7	3.55 (-3.08, 10.18)	0.294	< 0.001	93.5%	0.755
Overweight (25–29.9)	5	4.70 (1.79, 7.61)	**0.002**	< 0.001	98.6%	
Sex						
Male	2	4.46 (0.92, 7.99)	**0.013**	0.120	58.7%	0.671
Both	8	2.62 (0.17, 5.07)	**0.036**	< 0.001	97.7%	
Female	2	8.27 (-23.21, 39.76)	0.607	< 0.001	96.6%	
Health status						
Metabolic syndrome	3	2.46 (-2.63, 7.55)	0.343	0.022	73.9%	0.012
T2DM	5	10.50 (-0.57, 21.58)	0.063	< 0.001	85.6%	
Hyperlipidemic	2	4.41 (1.05, 7.76)	**0.010**	< 0.001	99.6%	
Hypertension	1	0.00 (-2.17, 2.17)	1.000	-	-	
NAFLD	1	-5.89 (-11.96, 0.18)	0.057	-	-	
Subgroup analyses of CLA on serum fasting insulin (mU/L)						
Overall effect	12	0.16 (-0.69, 1.02)	0.702	0.828	0.0%	
Trial duration (week)						
≥ 12	7	0.26 (-0.77, 1.30)	0.613	0.619	0.0%	0.737
<12	5	-0.04 (-1.57, 1.48)	0.951	0.718	0.0%	
Intervention dose (g/day)						
≥ 3	5	0.17 (-1.16, 1.50)	0.801	0.383	4.2%	0.802
<3	7	-0.08 (-1.53, 1.37)	0.912	0.891	0.0%	
Baselin BMI (kg/m^2^)						
Obese (> 30)	7	0.19 (-0.77, 1.16)	0.687	0.601	0.0%	0.891
Overweight (25–29.9)	5	0.05 (-1.81, 1.91)	0.956	0.725	0.0%	
Sex						
Male	2	3.62 (-6.42, 13.66)	0.480	0.351	0.0%	0.430
Both	8	-0.43 (-1.77, 0.90)	0.523	0.811	0.0%	
Female	2	0.55 (-0.57, 1.68)	0.335	0.552	0.0%	
Health status						
Metabolic syndrome	3	0.43 (-6.32, 7.20)	0.899	0.455	0.0%	0.998
T2DM	5	0.06 (-1.17, 1.30)	0.923	0.305	17.3%	
Hyperlipidemic	2	-0.02 (-5.20, 5.16)	0.994	0.824	0.0%	
Hypertension	1	-2.00 (-16.74, 12.74)	0.790	-	-	
NAFLD	1	-0.16 (-2.57, 2.25)	0.897	-	-	
Subgroup analyses of CLA on serum HbA1c (%)						
Overall effect	6	-0.03 (-0.17, 0.09)	0.567	0.038	57.6%	
Baseline HbA1c (%)						
< 6.5	3	0.05 (-0.22, 0.11)	0.529	0.004	82.3%	0.989
≥ 6.5	3	-0.05 (-0.40, 0.28)	0.748	0.841	0.0%	
Trial duration (week)						
≥ 12	3	0.02 (-0.03, 0.08)	0.385	0.587	0.0%	0.138
<12	3	-0.30 (-0.73, 0.12)	0.167	0.120	52.9%	
Intervention dose (g/day)						
≥ 3	3	0.02 (-0.03, 0.08)	0.385	0.587	0.0%	0.138
<3	3	-0.30 (-0.73, 0.12)	0.167	0.120	52.9%	
Baselin BMI (kg/m^2^)						
Obese (> 30)	4	-0.04 (-0.20, 0.11)	0.575	0.010	73.4%	0.859
Overweight (25–29.9)	2	-0.08 (-0.47, 0.31)	0.680	0.602	0.0%	
Sex						
Male	2	0.02 (-0.03, 0.08)	0.397	0.302	6.1%	0.144
Both	4	-0.25 (-0.61, 0.11)	0.178	0.157	42.4%	
Health status						
Metabolic syndrome	2	0.02 (-0.03, 0.08)	0.397	0.302	6.1%	0.006
T2DM	3	-0.05 (-0.40, 0.28)	0.748	0.841	0.0%	
NAFLD	1	-0.67 (-1.09, -0.24)	**0.002**	-	-	
Subgroup analyses of CLA on serum HOMA-IR						
Overall effect	11	0.34 (-0.11, 0.81)	0.140	< 0.001	78.7%	
Trial duration (week)						
≥ 12	5	0.23 (-0.56, 1.03)	0.563	< 0.001	87.1%	0.702
<12	6	0.43 (-0.18, 1.05)	0.171	0.007	68.4%	
Intervention dose (g/day)						
≥ 3	5	0.38 (-0.28, 1.05)	0.255	< 0.001	86.7%	0.931
<3	6	0.34 (-0.41, 1.10)	0.373	0.005	70.1%	
Baselin BMI (kg/m^2^)						
Obese (> 30)	7	0.27 (-0.26, 0.81)	0.321	< 0.001	81.0%	0.516
Overweight (25–29.9)	4	0.69 (-0.46, 1.86)	0.239	0.002	79.9%	
Sex						
Male	2	0.34 (-0.31, 1.00)	0.301	0.800	0.0%	0.017
Both	7	0.09 (-0.33, 0.52)	0.675	0.006	66.8%	
Female	2	1.27 (0.58, 1.97)	** < 0.001**	0.289	11.2%	
Health status						
Metabolic syndrome	1	0.23 (-1.87, 2.33)	0.830	-	-	0.371
T2DM	4	1.15 (-0.03, 2.34)	0.056	< 0.001	92.8%	
Hyperlipidemic	4	0.02 (-0.39, 0.44)	0.917	0.650	0.0%	
Hypertension	1	0.00 (-0.49, 0.49)	1.000	-	-	
NAFLD	1	-0.25 (-0.87, 0.37)	0.434	-	-	
Subgroup analyses of CLA on serum CRP (mg/L)						
Overall effect	7	0.00 (-0.45, 0.46)	0.976	0.489	0.0%	
Baseline CRP (mg/L)						
≥ 3	4	-0.52 (-1.47, 0.41)	0.273	0.636	0.0%	0.205
< 3	3	0.17 (-0.36, 0.72)	0.523	0.348	5.4%	
Trial duration (week)						
≥ 12	2	0.00 (-1.44, 1.44)	1.000	1.000	0.0%	0.974
<12	5	-0.02 (-0.61, 0.56)	0.931	0.245	26.5%	
Intervention dose (g/day)						
≥ 3	2	0.35 (-0.38, 1.10)	0.344	0.248	25.1%	0.159
<3	5	-0.36 (-1.03, 0.31)	0.292	0.746	0.0%	
Baselin BMI (kg/m^2^)						
Obese (> 30)	3	0.25 (-0.33, 0.84)	0.400	0.405	0.0%	0.166
Overweight (25–29.9)	3	-0.13 (-0.93, 0.67)	0.748	0.977	0.0%	
Normal (18.5–24.9)	1	-1.48 (-3.22, 0.26)	0.096	-	-	
Sex						
Male	2	0.35 (-0.38, 1.10)	0.344	0.248	25.1%	0.159
Both	5	-0.36 (-1.03, 0.31)	0.292	0.746	0.0%	
Health status						
T2DM	1	-0.19 (-1.15, 0.77)	0.699	-	-	0.861
Hyperlipidemic	5	0.06 (-0.59, 0.73)	0.841	0.285	20.4%	
NAFLD	1	-0.33 (-2.09, 1.43)	0.715	-	-	
Subgroup analyses of CLA on serum IL-6 (pg/ml)						
Overall effect	5	-0.44 (-0.86, -0.02)	**0.037**	0.078	52.3%	
Intervention dose (g/day)						
≥ 3	2	-0.32 (-0.66, 0.01)	0.064	0.101	62.8%	0.267
<3	3	-1.48 (-3.50, 0.53)	0.150	0.169	43.8%	
Baselin BMI (kg/m^2^)						
Obese (> 30)	3	-0.32 (-0.59, -0.05)	**0.019**	0.257	26.3%	0.059
Overweight (25–29.9)	1	-0.80 (-1.77, 0.17)	0.107	-	-	
Normal (18.5–24.9)	1	-3.81 (-6.87, -0.74)	**0.015**	-	-	
Sex						
Male	2	-0.32 (-0.66, 0.01)	0.064	0.101	62.8%	0.267
Both	3	-1.48 (-3.50, 0.53)	0.150	0.169	43.8%	
Health status						
T2DM	1	-0.80 (-1.77, 0.17)	0.107	0.023	73.6%	0.772
Hyperlipidemic	3	-0.41 (-0.94, 0.11)	0.121	-	-	
NAFLD	1	0.08 (-4.85, 5.01)	0.975	-	-	
Subgroup analyses of CLA on serum TNF-α (ng/l)						
Overall effect	4	0.26 (-0.16, 0.69)	0.232	0.141	45.0%	
Subgroup analyses of CLA on serum adiponectin (µg/ml)						
Overall effect	5	-0.12 (-2.41, 2.17)	0.918	< 0.001	98.7%	
Trial duration (week)						
≥ 12	1	-1.10 (-1.49, -0.70)	** < 0.001**	-	-	0.269
<12	4	0.24 (-2.10, 2.59)	0.838	< 0.001	95.3%	
Intervention dose (g/day)						
≥ 3	4	0.02 (-2.31, 2.35)	0.986	< 0.001	99.0%	0.513
<3	1	-3.63 (-14.30, 7.04)	0.505	-	-	
Baselin BMI (kg/m^2^)						
Obese (> 30)	4	0.02 (-2.31, 2.35)	0.986	< 0.001	99.0%	0.513
Overweight (25–29.9)	1	-3.63 (-14.30, 7.04)	0.505	-	-	
Sex						
Male	2	-0.59 (-1.32, 0.13)	0.110	0.708	0.0%	0.132
Both	2	1.84 (-1.57, 5.26)	0.290	0.268	18.4%	
Female	1	-1.10 (-1.49, -0.70)	** < 0.001**	-	-	
Health status						
T2DM	2	-1.10 (-1.49, -0.71)	** < 0.001**	0.643	0.0%	< 0.001
Hyperlipidemic	2	-0.59 (-1.32, 0.13)	0.110	0.708	0.0%	
Hypertension	1	2.40 (2.25, 2.54)	** < 0.001**	-	-	
Subgroup analyses of CLA on serum leptin (ng/ml)						
Overall effect	5	-1.69 (-1.80, -1.58)	** < 0.001**	0.451	0.0%	
Trial duration (week)						
≥ 12	3	-1.08 (-1.87, -0.30)	**0.007**	0.906	0.0%	0.208
<12	2	-1.81 (-2.63, -0.99)	** < 0.001**	0.282	13.8%	
Intervention dose (g/day)						
≥ 3	4	-1.68 (-1.79, -1.57)	** < 0.001**	0.475	0.0%	0.278
<3	1	-3.34 (-6.32, -0.35)	**0.028**			
Baselin BMI (kg/m^2^)						
Obese (> 30)	4	-1.68 (-1.79, -1.57)	** < 0.001**	0.475	0.0%	0.278
Overweight (25–29.9)	1	-3.34 (-6.32, -0.35)	**0.028**			
Sex						
Male	2	-1.35 (-2.94, 0.24)	0.097	0.812	0.0%	0.422
Both	2	-1.81 (-2.63, -0.99)	** < 0.001**	0.282	13.8%	
Female	1	-1.00 (-1.90, -0.09)	**0.030**	-	-	
Health status						
Metabolic syndrome	2	-1.35 (-2.94, 0.24)	0.097	0.812	0.0%	0.913
T2DM	2	-1.72 (-3.83, 0.39)	0.111	0.141	53.8%	
Hypertension	1	-1.70 (-1.81, -1.58)	** < 0.001**	-	-	
Subgroup analyses of CLA on serum MDA (mmol/l)						
Overall effect	3	-0.08 (-0.80, 0.62)	0.809	0.001	85.7%	
Subgroup analyses of CLA on serum ALT (IU/L)						
Overall effect	4	0.48 (-5.11, 6.07)	0.866	0.007	75.5%	
Subgroup analyses of CLA on serum AST (IU/L)						
Overall effect	4	2.54 (0.06, 5.01)	**0.044**	0.046	62.4%	

Abbreviations: *WMD* Weighted mean differences, *CI* confidence interval, *BMI* Body mass index, *FBG* Fasting blood glucose, *HbA1c* Hemoglobin A1c, *HOMA-IR* Homeostatic model assessment for insulin resistance, *CRP* C-reactive protein, *IL-6* Interleukin 6, *TNF-α* Tumor necrosis factor α, *MDA* Malondialdehyde, *ALT* Alanine transaminase, *AST* Aspartate transaminase

### Effect of CLA on CRP, TNF-α and IL-6

By analyzing seven overall effect sizes from five studies for CRP, four effect sizes from three studies for TNF-α, and five effect sizes from four studies for IL-6, it was revealed that CLA supplementation did not change CRP and TNF-α levels, significantly (for CRP WMD: 0.00 mg/L; 95%C: -0.45 to 0.46; *P* = 0.976) (Fig. 2E) (for TNF-α, WMD:0.26 ng/l; 95%CI: -0.16 to 0.69; *P* = 0.232) (Fig. 2G), but made a significant reduction in IL-6 levels (for IL-6, WMD: -0.44 pg/ml; 95%CI: -0.86 to -0.02; *P* = 0.037) (Fig. 2F). Additionally, a moderate degree of heterogeneity for both TNF-α (I^2^ = 45.0%), and IL-6 (I^2^ = 52.3%), was found among studies, whereas no between-studies heterogeneity was observed for CRP (I^2^ = 0.00%). Evaluating the results of subgroup analysis showed that CLA supplementation failed to decrease IL-6 levels significantly in overweight individuals (25 < BMI < 29.9), whereas lowered IL-6 levels in obese (BMI > 30) or normal BMI (18.5–24.9) participants (Table 3).

### Effect of CLA supplementation on adiponectin and leptin

Four studies with five effect sizes evaluated the effect of CLA supplementation on adiponectin and leptin. Pooled results from the random effects model demonstrated no significant alteration in adiponectin levels, whereas CLA supplementation diminished leptin levels, significantly (for adiponectin WMD: -0.12 µg/ml; 95%CI: -2.41 to 2.17; *P* = 0.918) (Fig. 2H), (for leptin WMD: -1.69 ng/ml; 95% CI:-1.80 to -1.58; *P* < 0.001) (Fig. 2I). Furthermore, a significant heterogeneity for adiponectin (I2 = 98.7%), and no heterogeneity for leptin (I^2^ = 0.00%) was observed among studies. Following the assessment of results in subgroup analysis, CLA supplementation failed to lower leptin levels in type 2 diabetic or metabolic syndrome patients, or male participants. Moreover, long-term CLA supplementation (≥ 12 weeks), or supplementation among female participants, hypertensive or type 2 diabetic patients, altered adiponectin levels (Table 3).

### Effect of CLA supplementation on AST and ALT

Four overall effect sizes from three studies for AST and ALT were assessed to reveal the effect of CLA on AST and ALT. It was shown that CLA supplementation did not affect ALT levels significantly (WMD: 0.48 IU/L; 95%CI: -5.11 to 6.07; *P* = 0.866) (Fig. 2K), but increased AST levels significantly (WMD: 2.54 IU/L; 95%CI: 0.06 to 5.01; *P* = 0.044) (Fig. 2L). In addition, a high heterogeneity for ALT (I^2^ = 75.5%) and a moderate for AST (I2 = 62.4%) were found among studies (Table 3).

### Effect of CLA supplementation on MDA

Three pooled overall effect sizes were analyzed and indicated that CLA supplementation failed to alter MDA levels significantly (WMD: -0.08 mmol/l; 95%CI: -0.80 to 0.62; *P* = 0.809) (Fig. 2J). Moreover, a significant degree of between-studies heterogeneity was seen (I^2^ = 85.7%) (Table 3).

### Sensitivity analysis

To assess the effect of each study on the overall effect size in this meta-analysis, we omitted each article. As a result, we did not observe any significant change in the overall results of FBG, Insulin, HbA1c, HOMA-IR, CRP, IL-6, TNF-α, Adiponectin, Leptin, MDA, ALT, and AST, following the CLA supplementation.

### Publication bias

**Fig. 3:**
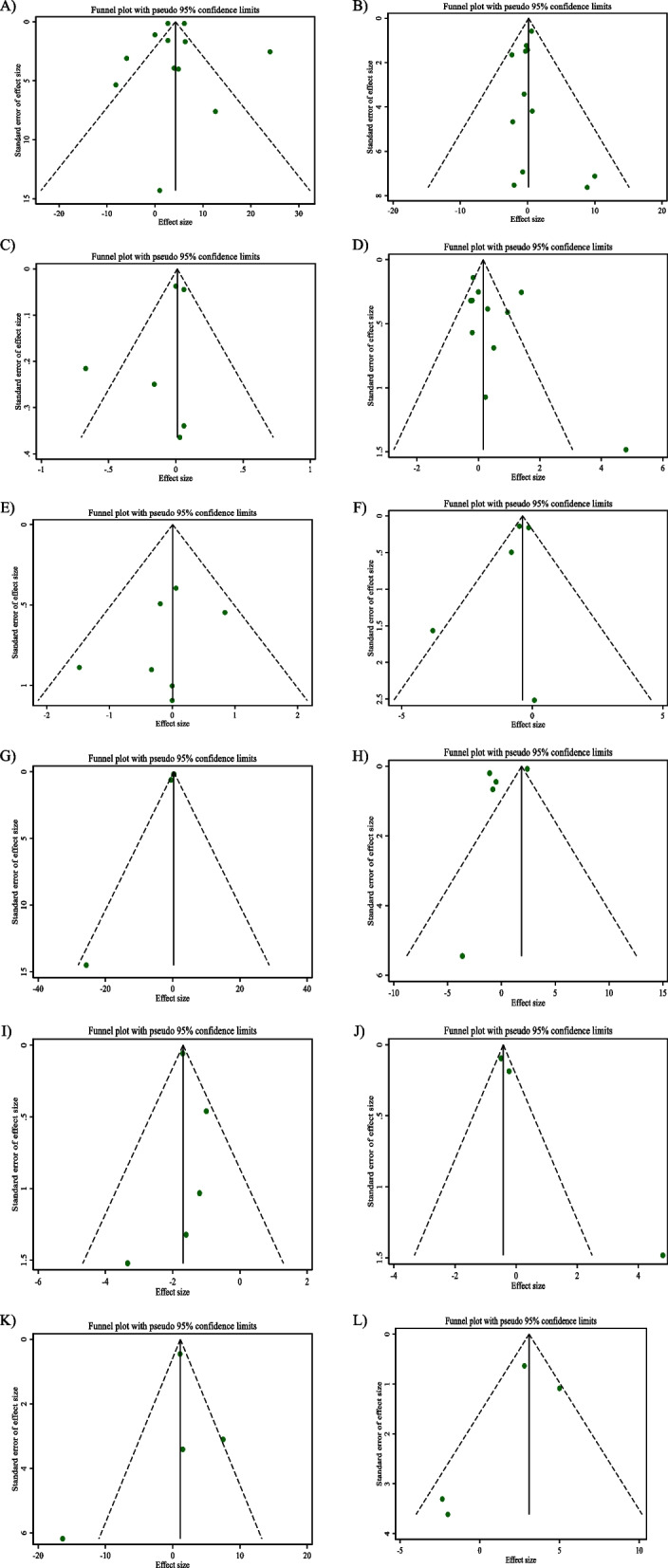
Funnel plots for the effect of CLA supplementation on **A** FBG (mg/dl); **B** Insulin (pmol/l); **C** HbA1c (%); **D** HOMA-IR; **E** CRP (mg/l); **F**; IL-6 (pg/ml); **G** TNF-α (pg/ml); **H** Adiponectin (ng/ml); **I** Leptin (ng/ml); **J** MDA (umol/l); **K** ALT (U/L); and **L**) AST (U/L)

Evaluating the results of Egger’s regression test indicated a significant publication bias in studies aimed to assess the effect of CLA supplementation on TNF-α, as an outcome (*P* = 0.040) (Fig. 3G).

### Non-linear dose–response analysis

**Fig. 4:**
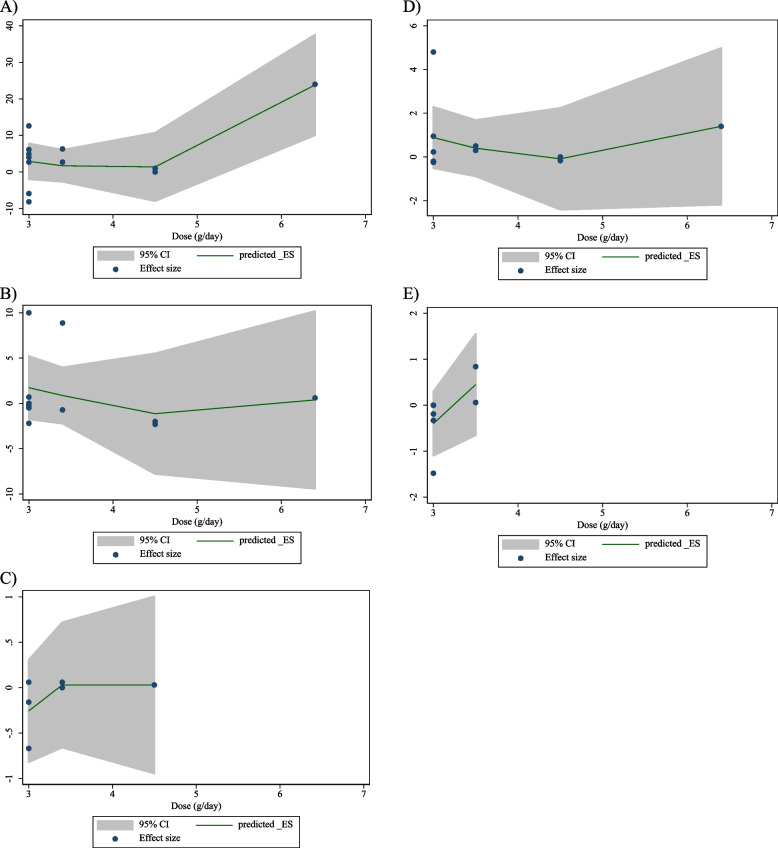
Non-linear dose–response relations between CLA supplementation and absolute mean differences. Dose–response relations between dose (mg/day) and absolute mean differences in on **A** FBG (mg/dl); **B** Insulin (pmol/l); **C** HbA1c (%); **D** HOMA-IR; and **E** CRP (mg/l)

**Fig. 5:**
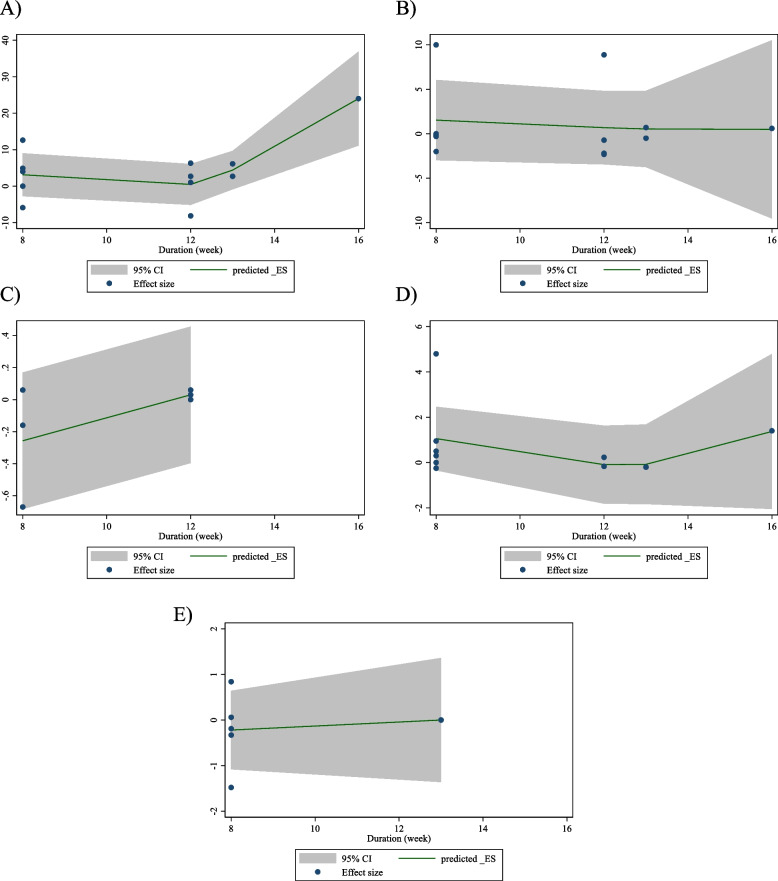
linear dose–response relations between CLA supplementation and absolute mean differences. Dose–response relations between dose (mg/day) and absolute mean differences in **A** FBG (mg/dl); **B** Insulin (pmol/l); **C** HbA1c (%); **D** HOMA-IR; and **E** CRP (mg/l)

**Fig. 6:**
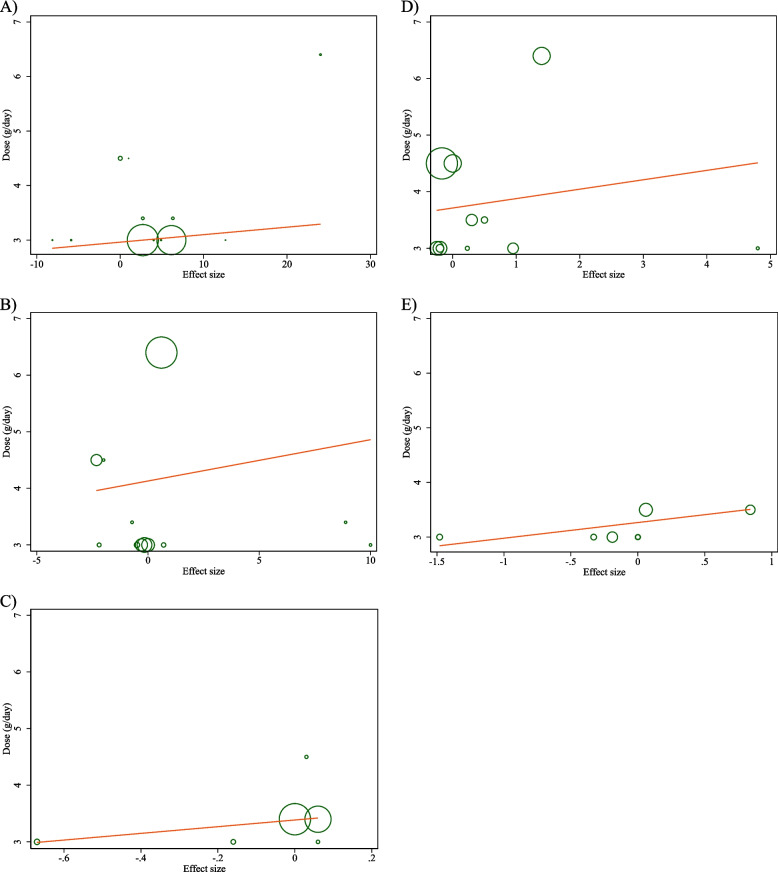
Non-linear dose–response relations between CLA supplementation and absolute mean differences. Dose–response relations between duration of intervention (week) and absolute mean differences in **A** FBG (mg/dl); **B** Insulin (pmol/l); **C** HbA1c (%); **D** HOMA-IR; and **E** CRP (mg/l)

The results of the non-linear dose–response analysis (Figs. 4 and 5) demonstrated a significant association between CLA supplementation and changes in FBG (*P* = 0.012) (Fig. 6A).

### Meta-regression analysis

**Fig. 7:**
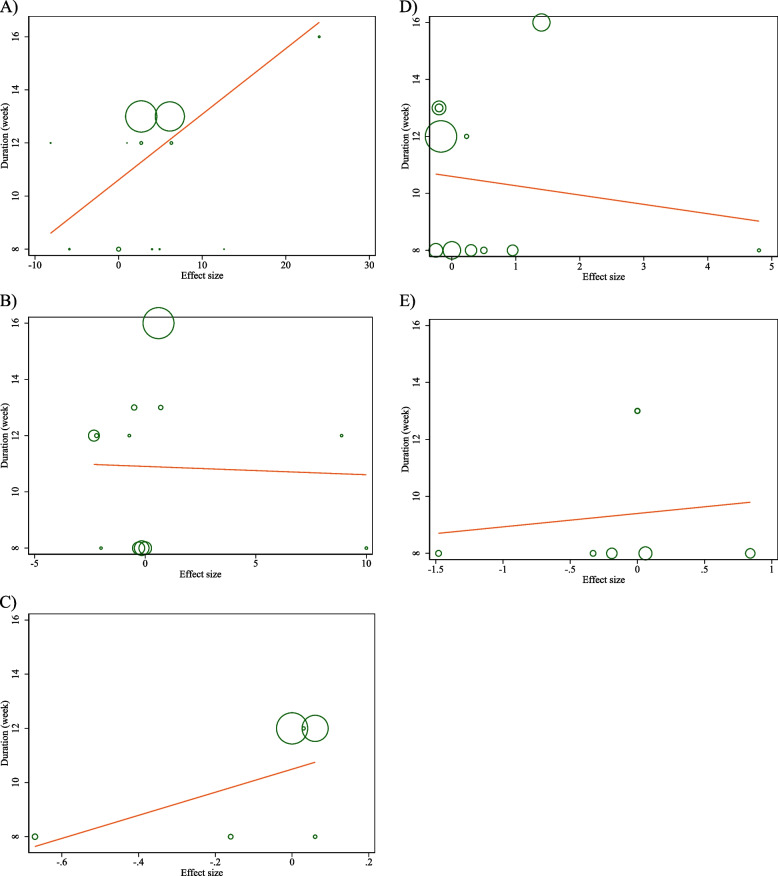
linear dose–response relations between CLA supplementation and absolute mean differences. Dose–response relations between duration of intervention (week) and absolute mean differences in **A**) FBG (mg/dl); **B** Insulin (pmol/l); **C** HbA1c (%); **D** HOMA-IR; and **E**) CRP (mg/l)

The outcomes of the meta-regression test revealed no significant association between the dose and duration of CLA supplementation and changes in levels of FBG, Insulin, HbA1c, HOMA-IR, CRP, IL-6, TNF-α, adiponectin, leptin, MDA, ALT, and AST (Figs. 5 and 7).

### GRADE analysis

The Grading of Recommendations, Assessment, Development, and Evaluations (GRADE) protocol was applied to assess the quality of the evidence for outcomes in this meta-analysis (Table 4). The studies examining the impact of CLA supplementation on FBG, HOMA-IR, TNF-α, Adiponectin, MDA, and ALT were considered to have a very low evidence quality. Furthermore, the articles evaluating the effect of CLA supplementation on HbA1C had a low quality evidence. On the other hand, the overall quality of the evidence showing the influence of CLA supplementation on Insulin, AST, IL-6, and CRP was upgraded to moderate. Lastly, high-quality evidence was observed for studies evaluating the effect of CLA supplementation on leptin (Table 4).

**Table 4:** GRADE profile of CLA supplementation for glycemic control, adipokine, cytokine, malondialdehyde and liver function enzymes in subjects with metabolic disorders

Outcomes	Risk of bias	Inconsistency	Indirectness	Imprecision	Publication Bias	Quality of evidence
FBG	No serious limitation	Very serious limitation ^a^	No serious limitation	No serious limitation	serious limitation	⊕ ◯◯◯ Very low
Insulin	No serious limitation	No serious limitation	No serious limitation	Serious limitation ^c^	No serious limitation	⊕ ⊕ ⊕ ◯ Moderate
HbA1c	No serious limitation	Serious limitation ^b^	No serious limitation	Serious limitation ^c^	No serious limitation	⊕ ⊕ ◯◯ Low
HOMA	No serious limitation	Very serious limitation ^a^	No serious limitation	Serious limitation ^c^	No serious limitation	⊕ ◯◯◯ Very low
CRP	No serious limitation	No serious limitation	No serious limitation	Serious limitation ^c^	No serious limitation	⊕ ⊕ ⊕ ◯ Moderate
IL-6	No serious limitation	Serious limitation ^b^	No serious limitation	No serious limitation	No serious limitation	⊕ ⊕ ⊕ ◯ Moderate
TNF-α	No serious limitation	Serious limitation ^b^	No serious limitation	Serious limitation ^c^	Serious limitation ^d^	⊕ ◯◯◯ Very low
Adiponectin	No serious limitation	Very serious limitation ^a^	No serious limitation	Serious limitation ^c^	No serious limitation	⊕ ◯◯◯ Very low
Leptin	No serious limitation	No serious limitation	No serious limitation	No serious limitation	No serious limitation	⊕ ⊕ ⊕ ⊕ High
MDA	No serious limitation	Very serious limitation ^a^	No serious limitation	Serious limitation ^c^	No serious limitation	⊕ ◯◯◯ Very low
ALT	No serious limitation	Very serious limitation ^a^	No serious limitation	Serious limitation ^c^	No serious limitation	⊕ ◯◯◯ Very low
AST	No serious limitation	Serious limitation ^b^	No serious limitation	No serious limitation	No serious limitation	⊕ ⊕ ⊕ ◯ Moderate

^a^There is high heterogeneity (I^2^ > 75) for FBG, HOMA-IR, adiponectin, MDA and ALT

^b^There is moderate heterogeneity (I^2^ > 40) for HbA1c, IL-6, TNF-α, and AST

^c^There is no evidence of significant effects of CLA supplementation on insulin, HbA1c, HOMA-IR, CRP, TNF-α, adiponectin, MDA and ALT

^d^There is a significant publication bias based on egger regression test for TNF-α (*P* = 0.04)

## Discussion

To our knowledge, this is the first GRADE-assessed systematic review and dose–response meta-analysis to evaluate the effects of CLA supplementation on glycemic control, adipokine, cytokine, MDA, and liver function enzymes in patients at risk of CVDs. Our study suggested that CLA supplementation was negatively associated with serum IL-6 and leptin and positively associated with FBG and AST, but generally, no associations with serum fasting insulin, HbA1c, HOMA-IR, CRP, TNF-α, adiponectin, MDA and ALT were observed. According to subgroup analyses, CLA decreased HbA1c in patients with NAFLD. Furthermore, in females, HOMA-IR levels increased. Moreover, among females with T2DM and in long-term intervention, adiponectin decreased. CLA also decreased adiponectin in hypertensive individuals.

CVDs and their risk factors are associated with 30% of all mortality worldwide [47]. Risk factors that are the leading causes of CVDs are dyslipidemia, high blood glucose, high blood pressure, obesity, and inflammation [48]. CLA, as a nutraceutical compound, has a beneficial effect on empowering the immune system, regulating glucose and lipid metabolism, and the CVD risk factors [49].

The present study failed to show improvement in glycemic profile after CLA supplementation. According to the data from animal studies, CLA may not have positive effects on the glycemic profile [50, 51]. In human studies, supplementation with CLA for 8 weeks did not cause a significant change in serum insulin and insulin resistance [52, 53]. In some studies conducted on obese individuals or individuals with metabolic syndrome, CLA increased blood glucose and insulin resistance [54–56]. The increase in blood glucose and insulin resistance due to the consumption of different isomers of 10-trans, 12-cis or 9-cis, 11-trans CLA has been reported [57]. While blood glucose increased in our study, there was no significant change in insulin sensitivity. The reason for the contradiction in the findings of these studies may be due to the difference in the responses of people [37, 58]. This difference may be related to the different types of diseases, participants' weight, the severity of the insulin resistance, the medicines taken by the patients, and the different amounts of CLA intake from the diet.

CLA has been shown to exert anti-inflammatory properties in animal models of disease [59]. However, CLA's anti-inflammatory effects must be clarified in human studies. Similar to our results, Aslani (2020) et al. suggested that 3.2 g daily consumption of CLA reduces inflammatory markers such as IL-6 serum levels, significantly [60]. Our recently published systematic review and meta-analysis of 42 studies showed that CLA increased CRP levels and decreased TNF-α and IL-6 levels [15]. Therefore, it seems that CLA can have both proinflammatory and anti-inflammatory roles. Since there is limited data about CLA's anti-inflammatory effects in patients at risk for CVDs, more RCTs are needed.

In agreement with our finding regarding the impact of CLA supplementation on leptin, Esmaeili Shahmirzadi et al. indicated that 6.4 gr/day CLA supplementation reduced serum leptin [61]. This decrease in serum leptin levels may be related to the significant reduction of adipose tissue and fat mass [62]. Our results were confirmed by one meta-analysis study [63] showed that short-term intervention of CLA supplementation (less than eight weeks) might decrease leptin in overweight subjects.

Over the past decades, it has been well-documented that ALT and AST, provoked immense interest as promising diagnostic biomarkers for various conditions, including CVDs and diabetes [64]. The present study found a non-significant increase in serum ALT and a significant increase in serum AST after CLA supplementation. Similar to our study, several previous studies did not see any effect on liver enzymes [21, 44, 45, 65]. Kadegowda et al. indicated that received CLA supplementation compared to the control group had an increase in liver weight due to hepatic steatosis [66]. Moreover, Wang et al. reported that a high dose of CLA supplementation can lead to fatty liver disease. This can be due to the compensatory pathway for reducing the fat accumulation in fat mass, instead of increasing lipogenesis and fat deposition in liver tissue [67]. Increasing AST as a measure of liver function due to CLA consumption (10-trans, 12-cis isomer) may suggest unwanted side effects. In a recent systematic review and meta-analysis by Haghighat et al., in the general population, ALT and AST levels did not change after CLA supplementation compared to the control group [24]. Based on these findings, the harmful properties of CLA supplementation on liver markers are more in participants at risk for CVDs.

The cardiovascular protective effects of CLAs are apparently mediated not only by CLAs themselves but also by their metabolites [68]. CLA intake improves blood pressure, a risk factor for CVD, by increasing adiponectin and endothelial nitric oxide synthase activity [69]. CLA activates 5’-adenosine monophosphate-activated protein kinase (AMPK) with concomitant increases in prostaglandin levels, sufficient to decrease lipids in adipocytes [70]. Moreover, the anti-steatotic effects of CLA may increase lipid utilization by peripheral tissues [71]. In animal models, CLA improves hepatic steatosis and restores liver triacylglycerol secretion and the fatty acid profile during protein repletion [72]. However, it should be noted that most protentional mechanisms of CLA supplementation on CVD risks are not based on patients at risk for CVDs. Therefore, more studies are needed to confirm our findings.

Our study had some limitations to be acknowledged. Subgroup analyses were not performed on some metabolic disease risk factors. In addition, no studies controlled for the diet, that might have effect on their results. Moreover, most studies did not evaluate extra CLA intake from diet. There were some strengths in this meta-analysis, including the publication bias not observe in this meta-analysis, and most of the included studies were double-blind, randomized and placebo-controlled trials, which increased the internal validity and decreased the biases.

## Conclusion

The findings of this meta-analysis supported the overall favorable effect of CLA supplementation on some of the adipokines and cytokines. CLA consumption was negatively associated with serum IL-6 and leptin. However, after CLA consumption, we found a significant increase in serum FBG and AST. It should be noted that the mentioned metabolic effects of CLA consumption were minor and may not reach clinical importance.

### Supplementary Information

**Additional file 1.**

## Data Availability

The datasets generated during and/or analyzed during the current study are available from the corresponding author on reasonable request.

## Abbreviations

ALT: Alanine aminotransferase
AST: Aspartate aminotransferase
BMI: Body mass index
CVDs: Cardiovascular diseases
CIs: Confidence intervals
CLA: Conjugated linoleic acid
CRP: C-reactive protein
FBG: Fasting blood glucose
GRADE: Grading of Recommendations, Assessment, Development and Evaluations
HbA1c: Hemoglobin A1c
HOMA-IR: Homeostatic model assessment for insulin resistance
IL-6: Interleukin 6
IQRs: Interquartile ranges
I^2^: I-squared statistic
MDA: Malondialdehyde
NAFLD: Non-Alcoholic Fatty Liver Disease
PRISMA: Preferred Reporting Items for Systematic Reviews and Meta-Analyzes
RCTs: Randomized controlled trials
SEs: Standard errors
TNF-α: Tumor necrosis factor alpha
WMD: Weighted mean differences
