# Supplementary material for: The effects of conjugated linoleic acid supplementation on glycemic control, adipokines, cytokines, malondialdehyde and liver function enzymes in patients at risk of cardiovascular disease: a GRADE-assessed systematic review and dose–response meta-analysis
Source: Nutr J. 2023 Oct 5;22:47. doi: 10.1186/s12937-023-00876-3 (PMC10552395; doi:10.1186/s12937-023-00876-3)
Supplement: Supplementary file 1 — Additional file 1. [file 12937_2023_876_MOESM1_ESM.docx]

January 2023

"Conjugated linoleic acid" OR "conjugated fatty acid" OR "bovic acid" OR "rumenic acid" OR "CLA"

Intervention OR "Intervention Study" OR "Intervention Studies" OR "controlled trial" OR randomized OR randomized OR random OR randomly OR placebo OR "clinical trial" OR Trial OR "randomized controlled trial" OR "randomized clinical trial" OR RCT OR blinded OR "double blind" OR "double blinded" OR trial OR "clinical trial" OR trials OR "Pragmatic Clinical Trial" OR "Cross-Over Studies" OR "Cross-Over" OR "Cross-Over Study" OR parallel OR "parallel study" OR "parallel trial"

| PubMed | ("Conjugated linoleic acid"[Title/Abstract] OR "conjugated fatty acid"[Title/Abstract] OR "bovic acid"[Title/Abstract] OR "rumenic acid"[Title/Abstract] OR "CLA"[Title/Abstract]) AND (Intervention[Title/Abstract] OR "Intervention Study"[Title/Abstract] OR "Intervention Studies"[Title/Abstract] OR "controlled trial"[Title/Abstract] OR randomized[Title/Abstract] OR random[Title/Abstract] OR randomly[Title/Abstract] OR placebo[Title/Abstract] OR "clinical trial"[Title/Abstract] OR Trial[Title/Abstract] OR "randomized controlled trial"[Title/Abstract] OR "randomized clinical trial"[Title/Abstract] OR RCT[Title/Abstract] OR blinded[Title/Abstract] OR "double blind"[Title/Abstract] OR "double blinded"[Title/Abstract] OR trial[Title/Abstract] OR trials[Title/Abstract] OR "Pragmatic Clinical Trial"[Title/Abstract] OR "Cross-Over Studies"[Title/Abstract] OR "Cross-Over"[Title/Abstract] OR "Cross-Over Study"[Title/Abstract] OR parallel[Title/Abstract] OR "parallel study"[Title/Abstract] OR "parallel trial"[Title/Abstract] OR OR[Title/Abstract]) | 3699 |
| --- | --- | --- |
| WOS | TOPIC: ("Conjugated linoleic acid" OR "conjugated fatty acid" OR "bovic acid" OR "rumenic acid" OR "CLA") AND TOPIC: (Intervention OR "Intervention Study" OR "Intervention Studies" OR "controlled trial" OR randomized OR randomized OR random OR randomly OR placebo OR "clinical trial" OR Trial OR "randomized controlled trial" OR "randomized clinical trial" OR RCT OR blinded OR "double blind" OR "double blinded" OR trial OR "clinical trial" OR trials OR "Pragmatic Clinical Trial" OR "Cross-Over Studies" OR "Cross-Over" OR "Cross-Over Study" OR parallel OR "parallel study" OR "parallel trial") | 2046 |
| Scopus | ( TITLE-ABS-KEY ( "Conjugated linoleic acid" OR "conjugated fatty acid" OR "bovic acid" OR "rumenic acid" OR "CLA" ) AND TITLE-ABS-KEY ( intervention OR "Intervention Study" OR "Intervention Studies" OR "controlled trial" OR randomized OR randomized OR random OR randomly OR placebo OR "clinical trial" OR trial OR "randomized controlled trial" OR "randomized clinical trial" OR rct OR blinded OR "double blind" OR "double blinded" OR trial OR "clinical trial" OR trials OR "Pragmatic Clinical Trial" OR "Cross-Over Studies" OR "Cross-Over" OR "Cross-Over Study" OR parallel OR "parallel study" OR "parallel trial" ) ) | 2286 |
| Cochrane | ("Conjugated linoleic acid" OR "conjugated fatty acid" OR "bovic acid" OR "rumenic acid" OR "CLA"):ti,ab,kw AND (Intervention OR "Intervention Study" OR "Intervention Studies" OR "controlled trial" OR randomized OR randomized OR random OR randomly OR placebo OR "clinical trial" OR Trial OR "randomized controlled trial" OR "randomized clinical trial" OR RCT OR blinded OR "double blind" OR "double blinded" OR trial OR "clinical trial" OR trials OR "Pragmatic Clinical Trial" OR "Cross-Over Studies" OR "Cross-Over" OR "Cross-Over Study" OR parallel OR "parallel study" OR "parallel trial"):ti,ab,kw | 485 |
| All |  | 8516 |
| Duplicates |  | 2182 |
| Remained |  | 6334 |
